# Correction: Synthesis of CuSe/PVP/GO and CuSe/MWCNTs for their applications as nonenzymatic electrochemical glucose biosensors

**DOI:** 10.1039/d4ra90067g

**Published:** 2024-06-10

**Authors:** Junaid Yaseen, Farhat Saira, Muhammad Imran, Mahvish Fatima, Hafiz Ejaz Ahmed, Muhammad Zeewaqar Manzoor, Momna Rasheed, Iqbal Nisa, Khalid Mehmood, Zahida Batool

**Affiliations:** a Institute of Physics, The Islamia University of Bahawalpur Pakistan zahida.batool@iub.edu.pk; b Nanoscience and Technology Development, National Center for Physics (NCP) Pakistan farhat.saira@ncp.edu.pk; c Chemistry Department, Faculty of Science, King Khalid University P.O. Box 9004 Abha 6141 Saudi Arabia; d Department of Physics, College of Science, Qassim University Buraydah 51452 Qassim Saudi Arabia; e Department of Physics, Government College University Faisalabad Pakistan

## Abstract

Correction for ‘Synthesis of CuSe/PVP/GO and CuSe/MWCNTs for their applications as nonenzymatic electrochemical glucose biosensors’ by Junaid Yaseen *et al.*, *RSC Adv.*, 2024, **14**, 6896–6905, https://doi.org/10.1039/D3RA06713K.

The authors regret that the name of one of the authors (Mahvish Fatima) and one of the affiliations (affiliation *d*) were incorrectly shown in the original article. The corrected author list and the corrected list of affiliations are as shown here.

The Royal Society of Chemistry apologises for these errors and any consequent inconvenience to authors and readers.

## Supplementary Material

